# Topical Semisolid Drug Product Critical Quality Attributes with Relevance to Cutaneous Bioavailability and Pharmacokinetics: Part I—Bioequivalence of Acyclovir Topical Creams

**DOI:** 10.1007/s11095-024-03736-9

**Published:** 2024-07-02

**Authors:** Y. H. Mohammed, S. N. Namjoshi, N. Jung, M. Windbergs, H. A. E. Benson, J. E. Grice, S. G. Raney, M. S. Roberts

**Affiliations:** 1 Therapeutics Research Centre, Frazer Institute, The University of Queensland, Brisbane, Australia; 2 School of Pharmacy, The University of Queensland, Woolloongabba, QLD 4102 Australia; 3 Institute of Pharmaceutical Technology, Goethe University, Frankfurt, Germany; 4 Curtin Medical School, Curtin University, Perth, WA Australia; 5 Office of Research and Standards, Office of Generic Drugs, Center for Drug Evaluation and Research, United States Food and Drug Administration, Silver Spring, MD USA; 6 School of Pharmacy and Medical Sciences, University of South Australia, Adelaide, Australia; 7 Therapeutics Research Centre, Basil Hetzel Institute for Translational Medical Research, The Queen Elizabeth Hospital, Adelaide, Australia

**Keywords:** bioavailability, bioequivalence, cutaneous pharmacokinetics, *in-vitro*, IVPT, Q3, topical

## Abstract

**Purpose:**

To develop a toolkit of test methods for characterizing potentially critical quality attributes (CQAs) of topical semisolid products and to evaluate how CQAs influence the rate and extent of active ingredient bioavailability (BA) by monitoring cutaneous pharmacokinetics (PK) using an *In Vitro* Permeation Test (IVPT).

**Methods:**

Product attributes representing the physicochemical and structural (Q3) arrangement of matter, such as attributes of particles and globules, were assessed for a set of test acyclovir creams (Aciclostad® and Acyclovir 1A Pharma) and compared to a set of reference acyclovir creams (Zovirax® US, Zovirax® UK and Zovirax® Australia). IVPT studies were performed with all these creams using heat-separated human epidermis, evaluated with both, static Franz-type diffusion cells and a flow through diffusion cell system.

**Results:**

A toolkit developed to characterize quality and performance attributes of these acyclovir topical cream products identified certain differences in the Q3 attributes and the cutaneous PK of acyclovir between the test and reference sets of products. The cutaneous BA of acyclovir from the set of reference creams was substantially higher than from the set of test creams.

**Conclusions:**

This research elucidates how differences in the composition or manufacturing of product formulations can alter Q3 attributes that modulate myriad aspects of topical product performance. The results demonstrate the importance of understanding the Q3 attributes of topical semisolid drug products, and of developing appropriate product characterization tests. The toolkit developed here can be utilized to guide topical product development, and to mitigate the risk of differences in product performance, thereby supporting a demonstration of bioequivalence (BE) for prospective topical generic products and reducing the reliance on comparative clinical endpoint BE studies.

## Introduction

Topical semisolid products are designed to deliver an active pharmaceutical ingredient (API) to a local site of action in the skin or underlying tissues for the treatment of dermatological, musculoskeletal and other conditions [1, 2]. The formulation components and composition, as well as the physicochemical and structural (Q3) arrangement of matter in the dosage form can be critical determinants of product performance, because these product quality attributes can modulate the rate and extent to which the API becomes available at the site(s) of action in the skin. The arrangement of matter in a semisolid product (which can be described by a comprehensive profile of Q3 attributes) [3, 4] is influenced by the product composition and by manufacturing process parameters [4]. The Q3 attributes of topical semisolid products describe the arrangement of matter in these dosage forms and represent the physicochemical environment and structural machinery that control processes within the dosage form, thereby governing how the product performs in any given situation. Thus, differences in Q3 attributes may alter product performance. By contrast, consistent control of Q3 attributes for a product can help ensure consistent product performance. It is, therefore, rational to expect that orthogonal evidence from a collection of relevant product characterization test which systematically demonstrate a well-matched profile of relevant Q3 attributes for a test product (e.g., a prospective generic product) and a reference standard topical semisolid product can mitigate the risk of differences in product performance, and thereby, support a demonstration of BE [5]. Improving the efficiency of product development and of the regulatory assessment of BE for prospective generic drug products has a powerful social impact because the availability of generic drug products from multiple manufacturers stabilizes the supply chain and reduces the risk of drug shortages, increases market competition which typically lowers the cost of medicines, and enhances patient access to high quality, safe, effective, and affordable medicines [6].

In the case of topical semisolid drug products, well-defined critical quality attributes (CQA) are essential to effectively characterize the products, and to compare the Q3 attributes of test and reference standard products in a manner that can support a demonstration of BE [4, 7, 8]. The quality attributes (QAs) that can influence product performance include solubility, particle size, pH, rheological properties, globule size distribution, polymorphic states, type of emulsion and oil to water phase ratio [4, 8, 9]. Also, manufacturing process variables, such as homogenisation speed and time, can alter globule size, homogeneity, and viscosity. As an example, viscosity differences may have the potential to alter the rate of evaporation for a product, and thereby alter its composition, solubility, and thermodynamic activity profile, ultimately altering the rate and extent of drug delivery [7, 10]. Other CQAs that can be highly dependent on the manufacturing process parameters include rheology, product uniformity, precipitation, and recrystallization or stability of APIs [11]. In this work, we have developed a toolkit to characterize the arrangement of matter (i.e., to characterize a profile of Q3 attributes) in a topical semisolid dosage form, and have explored the relationship between the arrangement of matter (the Q3 attributes) and the underlying matter (the components and composition) for a series of related products [8].

*In vitro* permeation test (IVPT) studies using ex vivo human skin are useful to compare the rate and extent of bioavailability (BA) and the cutaneous pharmacokinetics (PK) of compounds delivered into and through the skin from topical products, assuming appropriate validation of protocols and techniques [12–15]. IVPT studies can be more efficient and less expensive compared to performing a comparative clinical endpoint BE study [16]. They are used routinely to characterize differences in percutaneous absorption that may result from changes in the composition of the product formulation, which can be one of the most critical factors affecting BA and BE. The IVPT studies conducted as part of this work utilized a consistent protocol throughout. Certain key study parameters (e.g., topical semisolid product treatment groups, and dose amount per area) were coordinated and harmonized with comparable IVPT studies performed by other research groups using the same set of acyclovir cream products. Certain differences in protocol parameters between research groups were intentionally introduced to evaluate the effects of those differences (e.g., diffusion cell apparatus, skin preparation) and to thereby facilitate assessments of the relative influence of different protocol parameters on the outcomes of an IVPT study.

In this study, we evaluated multiple Q3 attributes, and used multiple test methods to characterize these Q3 attributes, for a set of pharmaceutically equivalent acyclovir cream products. Topical acyclovir cream, 5% was selected as a model drug product because it represented a complex topical semisolid drug product that (like many other products) might not be feasible to develop as a generic product in the United States utilizing a comparative clinical endpoint BE study. Also, it was a product for which different pharmaceutically equivalent variations existed in different parts of the world, and there was published literature [17] to suggest that these different acyclovir cream, 5% products sold in different parts of the world may not have equivalent BA of acyclovir. This allowed us to characterize and compare Q3 attributes among acyclovir creams with comparable cutaneous PK profiles, or with different cutaneous PK profiles, and to evaluate what similarities or differences in Q3 attributes might be associated with similar or different cutaneous PK profiles.

It was not trivial to conceive what aspects of a cream’s arrangement of matter might have the potential to influence the rate and extent of acyclovir delivery to the site of action, and then to describe specific concepts for what Q3 attributes would be associated with those corresponding failure modes for BE, and further, to then develop specific test methods to assess product quality attributes that would either directly or indirectly characterize the Q3 attributes of interest. A further challenge was to then design studies that would elucidate whether similarities or differences in the Q3 attributes among different acyclovir creams correlated with the cutaneous PK of acyclovir from the respective acyclovir cream products. By strategically and systematically addressing these research questions and challenges, we successfully developed a toolkit of test methods for the characterization of Q3 attributes in topical semisolid products, and related these Q3 characterizations to product performance based upon cutaneous PK.

## Materials and Methods

### Materials

Acyclovir cream formulations containing 5% w/w Acyclovir from multiple countries were used in this study. Acyclovir topical cream, 5% marketed in the United States (Zovirax® U.S.) was considered as the reference standard [18]. This product was compared to other pharmaceutically equivalent Zovirax® acyclovir creams marketed in the United Kingdom (Zovirax® U.K.), and Austria (Zovirax® Austria), as well as two other pharmaceutically equivalent acyclovir creams (Aciclostad® and Aciclovir 1A Pharma®) marketed in Austria. For the purposes of this study, the three Zovirax® branded creams were considered a reference set of products, and the two Aciclostad® and Aciclovir 1A Pharma® creams were considered a test set of products. Table 1 below lists the composition of all the creams tested.

**Table 1:** **C**omposition of Acyclovir creams

Cream Name	Zovirax® (U.S.)	Zovirax® (U.K.)	Zovirax® (Austria)	Aciclostad® (Austria)	Aciclovir 1A Pharma® (Austria)
ACV concentration	1 g = 50 mg ACV	1 g = 50 mg ACV	1 g = 50 mg ACV	1 g = 50 mg ACV	1 g = 50 mg ACV
Other Ingredients:	Cetostearyl alcohol Mineral oil Poloxamer 407 Sodium lauryl sulfate Water White petrolatum	Poloxamer 407 Cetostearyl alcohol Sodium Lauryl Sulfate White soft paraffin Liquid paraffin Purified water Arlacel 165 (containing glycerol monostearate and polyoxyethylene stearate) Dimethicone 20	Cetostearyl alcohol White vaseline Liquid paraffin Poloxamer Sodium dodecyl sulfate Dimethicone Glycerol monostearate Macrogol-100-stearate Purified water	White vaseline Liquid paraffin Macrogol stearate Dimethicone Purified water	White vaseline Viscous paraffin Glycerol monostearate Polyoxyethylene stearate Dimethicone Purified water

All the reagents used were analytical grade. Acyclovir drug standard was obtained from Sigma-Aldrich, product no: P500254, for development of the HPLC method. Acetonitrile (batch no: 15050300) was supplied by RCI Labscan Ltd. (Port Adelaide, South Australia) and ammonium acetate was obtained from AnalaR MERCK Pty. Ltd, Victoria Australia. Phosphate buffered saline (PBS) used in the IVPT experiments was prepared by dissolving 1 pouch of PBS powder, pH 7.4 obtained from Sigma (Lot number: S2BJ4837U) in 1 L purified water.

### Analysis of Particle Size and Morphology

Acyclovir drug particles suspended in the cream base were visualised using simple optical microscopy, non-contact optical profilometry and contact 3D profiling using Atomic Force Microscopy (AFM). Optical microscopic images were utilized for particle size measurements. Cryo-scanning electron microscopy (cryo-SEM) and confocal Raman microscopy (CRM) were employed for morphological and spectral evaluation of the products.

For optical microscopy, cream samples were applied onto glass slides and spread evenly in a thin layer using a cover slip. Images were acquired using a Zeiss Primostar optical microscope (Carl Zeiss Microscopy GmbH, 07745 Jena, Germany) with a Zeiss AxioCam ERc 5S. Four microscopy images (approximately 25 particles per image) were acquired for each sample (40 × magnification). In each image, particles were manually counted and measured using AxioVision software (Release 4.8.2) to obtain particle size information that was subsequently imported into Excel to generate particle size distribution plots. Particle size was determined by the projected area diameter method [12, 19, 20].

An Olympus LEXT OLS4100 laser scanning digital microscope equipped with a BF Plan Semi-apochromat 5 × and 10 × objective and a LEXT-dedicated plan apochromat 20x, 50 × and 100 × objective was used to acquire 3D images in a non-contact manner. The sample preparation included applying the cream samples in a thin layer to a glass slide. LEXT Olympus image analysis software was used to construct 3D height maps and topographic images.

Contact mode profilometry was conducted using an Asylum Research MFP-3D Bio AFM in tapping mode. A 10 nm tip radius HA_NC cantilever from NT-MDT was used to carefully map the topography of the surface at 50 × 50 µm, 20 × 20 µm, 5 × 5 µm and 2 × 2 µm scan area. A scan frequency of 0.6 Hz and a set point of 746 mV were used to scan the sample. The sample preparation involved the above-described spreading technique followed by a 12–14 h drying period to render the surface of the sample to be imaged in contact mode. The acquired images were analysed using Igor Pro 6.36 software to generate 2D pseudocolored height and amplitude maps as well as 3D contour images.

### Examination of Globules and Chemical Composition of the Cream Microstructure

The cream samples were visualised by SEM and CRM, with particular emphasis on the visualization of globules and spectral differentiation where possible.

Morphological examination of the internal microstructure of the acyclovir creams in their native form was carried out using a JEOL scanning electron microscope (JSM7100F, JEOL, USA), equipped with a secondary electron detector. For the cryo-SEM, the cream samples were loaded on the cryo-specimen holder and cryo-fixed in slush nitrogen (-210 °C), then quickly transferred under vacuum to the cryo-preparation chamber in the frozen state. The frozen cream samples were fractured using an in-built fracture blade and then sputter-coated with platinum for 120 s at 10 mA. The coated samples were moved to the imaging chamber maintained at -145 °C, equipped with an anti-contaminator which was maintained at -194 °C. The imaging was undertaken at a voltage of 2 kV by collecting the secondary electron signal.

For particle morphological and spectral analysis with CRM, the cream samples were spread evenly on a CaF_2_ slide using a cover slip. The chemical composition of each cream was determined by an alpha300 R^+^ confocal Raman microscope (WITec GmbH, Ulm, Germany) coupled to a diode laser with a wavelength of 785 nm. The laser power was adjusted to 20 mW before a 50 × objective with a numerical aperture of 0.8. Raman spectra were recorded in a range of 400—1780 cm^−1^ with a resolution of 4 cm^−1^. Bright field images of the creams were recorded, and Raman analysis of the indicated region was performed. Single spectra were obtained by using an acquisition time of 10 s and 10 accumulations. A pinhole of 100 µm was chosen to reject signals from out-of-focus-regions. Scans in XY-direction were recorded with a step size of 0.5 µm and an acquisition time of 0.2 s per spectrum. On all recorded Raman spectra, a cosmic ray removal and a background subtraction were performed. For image scans additional cluster analysis followed by basis analysis were performed to create false-colour images. All processing steps of Raman spectral data sets were performed using the Project FOUR software (WITec GmbH, Ulm, Germany).

### Determination of Formulation pH

The pH of all products was measured using a Mettler-Toledo In lab micro pH probe following a 3-point calibration (pH 4, 7, 10). The acyclovir creams were transferred into micro-centrifuge tubes with minimal shear ensuring that the microstructure of the cream was retained. The pH micro probe was inserted into the cream and the pH recorded after the reading had stabilized. The micro probe was removed, wiped, and washed with Milli Q water after each measurement. The procedure was conducted at least in triplicate for each cream.

### Determination of the Polymorphic Form of Acyclovir in the Creams

The polymorphic state of the API and the chemical composition of the acyclovir cream products was analysed by complementary techniques including X ray powder diffraction (XRPD), CRM and Differential scanning colorimetric analysis (DSC).

X ray diffractograms were collected on a Bruker D8 Advance with DaVinci design, LYNXEYE scintillation detector and Cu Ka radiation (l = 1.5405 Å), voltage 40 kV and current 40 mA. The data was analysed by DiffracSuite™ (V2.2) software. A cup shaped sample holder was used to accommodate a higher volume of sample to maximise the orientations of the crystals available for diffraction. Diffractogram collection was carried out over 2u range of 4 – 40, at an increment of 0.0114 at 1 s per step. The sample holder was in rotation to get the average diffractogram of the sample.

CRM was performed on acyclovir creams as described above. Single Raman spectra of the drug crystals were acquired on multiple spots and compared to spectra of all polymorphic forms of acyclovir to determine the chemical identity. As references, Raman spectra of polymorphic forms I, II and V were recorded with an integration time of 5 s and 10 accumulations. The commercial acyclovir form V was obtained via Fargon (Lot number: 14A22-B06-290543), polymorphic forms I and II were prepared as described by Lutker et al. [20], Raman spectra of polymorphic forms III, IV and VI were used as reference from Lutker et al. [20].

All DSC-TGA measurements were made on a TGA/DSC 2 STAR system (Mettler-Toledo, Greifensee, Switzerland) that was calibrated using blank alumina crucibles. A known amount of acyclovir standard or acyclovir cream sample (40 mg) was placed in an alumina pan and heated at a rate of 10 °C/min from room temperature to 600 °C.

### Determination of Water Content in Acyclovir Creams

Several solvents were tested to solubilize the different creams. A 2:1 methanol/chloroform mixture was found to be suitable to adequately dissolve the cream matrix. 30 mL of 2:1 methanol: chloroform mixture was added to the titration flask. It was neutralized with Karl Fischer reagent to the electrometric end point. A known quantity of sample (weighed accurately) was transfer into a titration flask for 1 min and titrated with Karl Fischer reagent to the electrometric end point. The volume of Karl Fischer reagent consumed was recorded.

### Determination of Loss of Water from Formulations

A novel method was developed to determine water loss from cream products, based on the measurement of TEWL using an AquaFlux AF 200 instrument equipped with an AquaFlux software Version 8 (Biox, UK). 15 mg of test cream was spread on a pre-weighed glass slide (application area 2.5 × 2.5 cm) using a cover slip, and the cream weight was recorded. The AquaFlux was applied using a closed mount system (measurement area 1.33 cm^2^) and TEWL readings taken at 0, 10, 20, 40 and 60. A gravimetric method was also used whereby the weight loss (determined using a Shimadzu AUW220D microbalance) due to the evaporation of ingredients was also measured on separate samples at the same time points. All measurements were taken at room temperature (25 °C ± 1 °C), and at skin surface temperature (32 °C ± 1 °C). Four replicates of each cream product were analysed, and data plotted as mean water loss (mg) against time.

### Quantification of Acyclovir

A Shimadzu HPLC system equipped with a binary solvent pump, auto sampler, photodiode array detector, thermostatted column compartment and LC solutions chromatographic software was used to measure the concentration of acyclovir. Isocratic flow at 1 mL/min was used with a mobile phase consisting of a mixture of 10 mM ammonium acetate and 5% v/v acetonitrile at pH 6.5. Chromatographic separation was performed with a Phenomenex Luna (5μ) C18 column (4.6 × 150 mm) maintained at 25 °C, after injection of 10 μL samples, with detection at 254 nm. 50 µL of the acyclovir standard or unknown solution (i.e., receptor solution or other extract) was added to 25 µl of the internal standard solution (85 µg/mL theophylline in MQ water) and vortexed to mix. The calibration curve range of acyclovir was 12 ng/mL to 100 μg/mL.

### *In-vitro* Skin Permeation of Acyclovir from Acyclovir Cream, 5% Products

#### Human Skin Preparation

Full thickness human skin samples obtained from patients (26–48 years old females) undergoing abdominoplasty at Brisbane (QLD) hospitals were refrigerated immediately after elective surgery. Sampling was approved by the Metro South and University of Queensland Human Research Ethics Committees (Approval number: 2008001342) and was conducted in compliance with the guidelines of the National Health and Medical Research Council of Australia and FDA RIHSC. Epidermal sheets were obtained by first removing subcutaneous fat by dissection, then immersion of full thickness skin in water at 60 °C for 1 min, after which the epidermis was teased off the dermis [21]. The epidermis was air dried, then placed in a zip-lock bag and stored at –20 °C until required.

#### *In Vitro* Permeation Test (IVPT) Studies

*In vitro* permeation test studies across human epidermis were performed in Pyrex glass Franz-type diffusion cells (exposed skin area 1.33 cm^2^; receptor volume approximately 3.5 mL). Epidermal membranes were placed between the donor and receptor compartments and allowed to equilibrate with the receptor solution (PBS pH 7.4 and 0.01% sodium azide) that was stirred continuously with a magnetic stirrer bar. The receptor compartment was immersed in a water bath at 37 ± 0.5 °C and maintained the skin surface temperature at approximately 32 °C, as measured by an infrared thermometer. PBS was placed in the donor compartment and the resistance across the epidermis was measured with a digital multimeter to determine membrane integrity. Membranes exhibiting an electrical resistance of less than 20 kΩ were rejected from the study [22]. The receptor was then refilled with approximately 3.5 mL (measured accurately) of fresh pre-warmed receptor solution.

Pre-weighed amounts of acyclovir cream, 5% (approximately15 mg/cm^2^) using Zovirax® branded products marketed in the US, UK, and Austria, as well as other products (Aciclostad® and Aciclovir 1A Pharma®) were applied to the surface of the epidermis by spreading evenly with a 1 mL syringe plunger that was weighed before and after the spreading procedure. 200 μL samples of the receptor solution were withdrawn at various times over a 48-h period and replaced with equal volumes of fresh pre-warmed (37 °C) PBS (pH 7.4) containing 0.01% sodium azide. Acyclovir concentrations in the receptor samples were analysed by HPLC. A total of 9 replicates from 3 skin donors were used for each acyclovir cream product. The cumulative amount of acyclovir permeated through the epidermis (μg/cm^2^) versus time (h) was plotted and the flux through the epidermis was determined from the slope of the plot of cumulative amount versus time and expressed as μg/cm^2^/h.

### Statistical Analysis

Average particle size and d10, d20, d90, loss of water and volatiles, as well as the rate and extent of acyclovir delivery were plotted using GraphPad Prism 7.0. GraphPad was also used to compare differences between the formulations tested. The cumulative amounts (µg/cm^2^) of acyclovir in the receptor chamber were determined over the entire period of testing and compared between the different products by an unpaired t test (GraphPad Prism 7.0). Flux (µg/cm^2^/h) was calculated at each time point by measuring the change in permeated amount from each sampling time to the next. An unpaired t test was used to compare the difference between acyclovir fluxes from the different products tested. A result was considered significant with P < 0.05.

## Results

### Particle Size Analysis

**Fig. 1:**
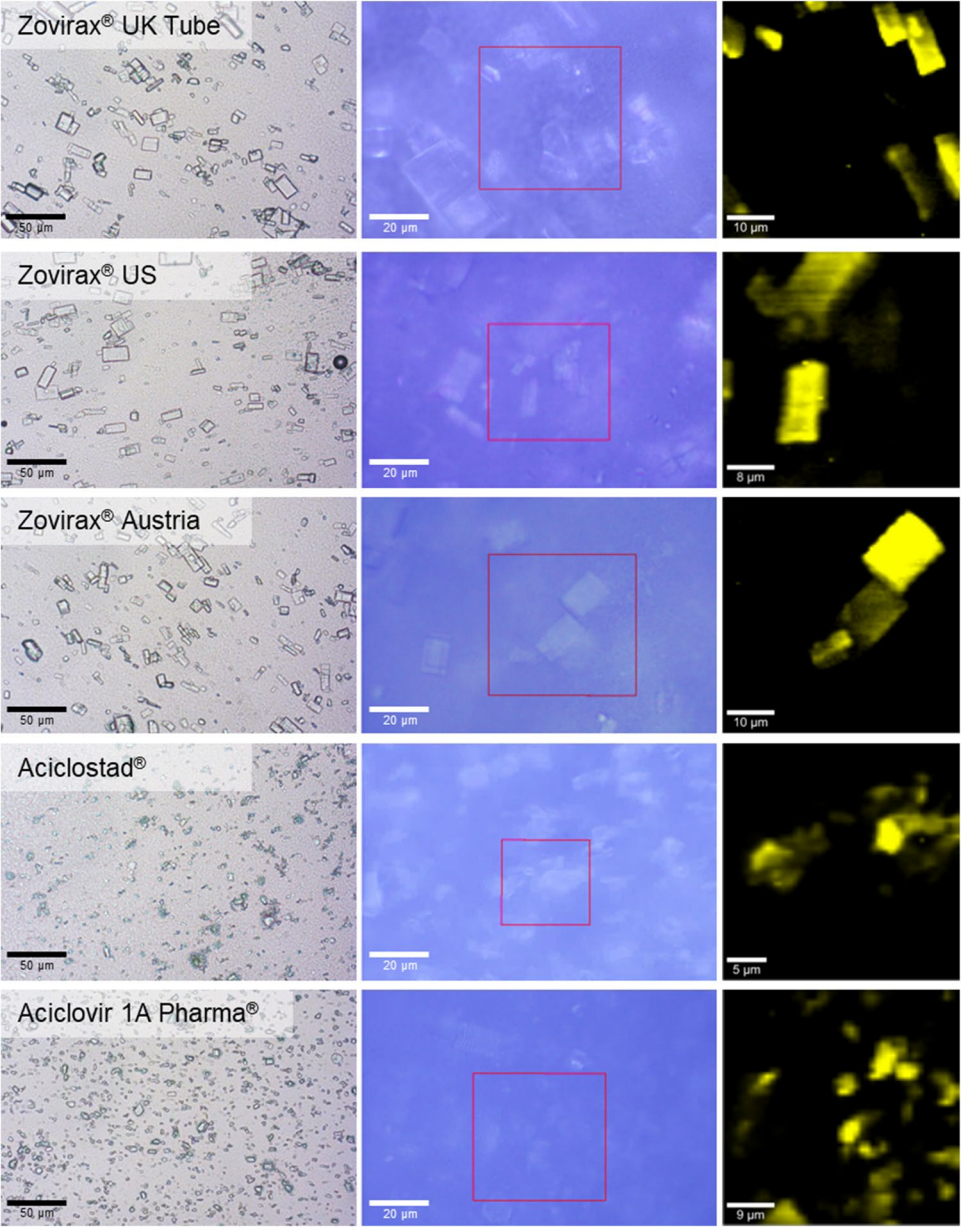
Optical Microscopic Images of All the Creams (Panel 1) and the Associated Confocal Raman Images of Acyclovir Crystals (Yellow) and Cream Base (Black).

The morphology of the drug crystals was investigated in all acyclovir cream, 5% products. Images acquired by optical microscopy revealed that acyclovir crystals were uniformly suspended in the cream base of the reference set of Zovirax® products and that their shape resembled uniform, thin, rectangular plates. However, drug crystals in the test set of products, Aciclostad® and Aciclovir 1A Pharma**®**, exhibited an irregular shape (Fig. 1). All creams had projected area diameter particle sizes < 100 µm, with d50 ranging from 2.90 µm to 8.3 µm (Table 2). **S**imilar particle size results were also obtained from CRM, and the differences in crystal shape between the reference and test sets of acyclovir creams were also evident. Figure 1 illustrates the particle size distribution with optical microscopy and CRM.

**Table 2:** Particle size distribution of acyclovir in all 5% acyclovir creams determined using optical microscopy. d10, d20 and d90 represent the percent (10%, 20%, and 90%, respectively) of the population of particles whose diameter is smaller than the indicated size

Products	d10 (µm)	d50 (µm)	d90 (µm)
Aciclostad®	2.72	4.38	9.72
Acyclovir 1 A Pharma®	1.91	2.90	6.24
Zovirax® US	3.63	6.92	16.60
Zovirax® UK	3.52	6.22	19.35
Zovirax® Austria	4.61	8.26	18.34

Contact and non-contact 3D profiling were both able to highlight the surface structure in 3D. Both techniques were useful for examining topographical details, but their usefulness would be limited in systems such as Acyclovir 5% creams.

### Morphological Examination and Spectral Identification of Creams

**Fig. 2:**
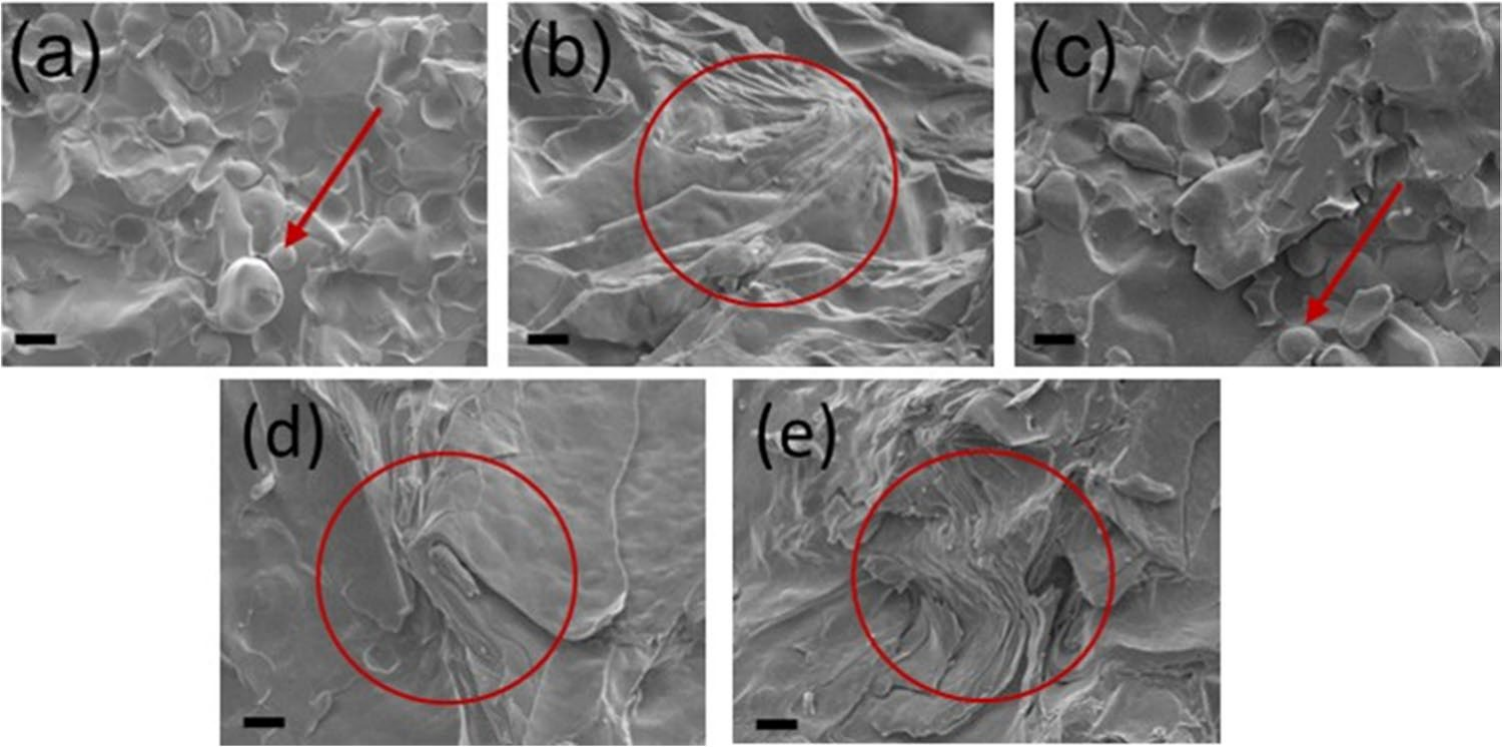
Cryo-SEM images showing the internal microstructure of the acyclovir creams (**a**) Acyclovir 1A Pharma®, (**b**) Zovirax® Austria, (**c**) Aciclostad®, (**d**) Zovirax® UK and (**e**) Zovirax® US. The cream samples were imaged at 10,000 × magnification and the bar represents 1 μm.

Figure 2 shows the microstructural differences (globules, crystals, and the cream fabric) between the different creams gained from cryo-SEM. Oil globules (red arrows) were observed in the test set of products, Aciclostad® and Aciclovir 1A Pharma® (Fig. 2 a and c). The reference set of Zovirax® products exhibited a tightly organized lamellar microstructure which is formed by alternating layers of surfactants and lipids (Fig. 2 b, d, and e).

**Fig. 3:**
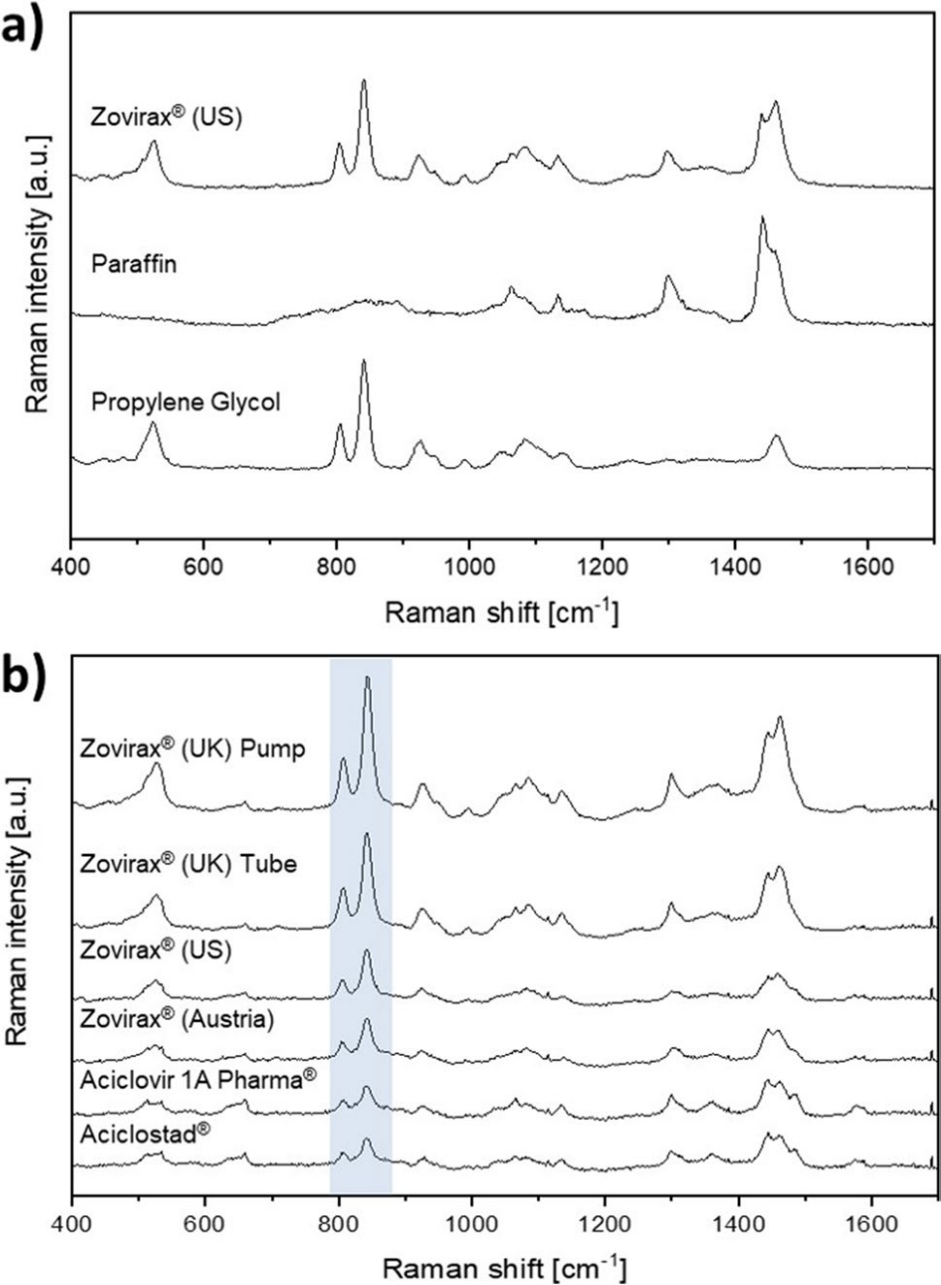
(**A**) Identification of excipients of Zovirax® US based on spectral attributes of the cream base. (**B**) Comparison of Raman spectra of the cream base of all formulations. Highlighted peaks are associated to propylene glycol.

Raman spectra from the cream base of Zovirax® US can clearly be assigned to paraffin and PG as shown in Fig. 3(A). The spectrum of Zovirax® US comprises mainly spectral features of PG with the characteristic double peak at 840 cm^−1^ as well as some peaks related to paraffin (1280 cm^−1^, 1450 cm^−1^). Raman spectra of the cream base the reference set of Zovirax® creams, and the test set of creams, show PG as the most prominent excipient. The differing intensities of the compound, as indicated by the highlighted area in Fig. 3(B), are likely due to varying amounts of PG incorporated in the formulations.

### Analysis of Different Acyclovir Polymorphs in the Creams

**Fig. 4:**
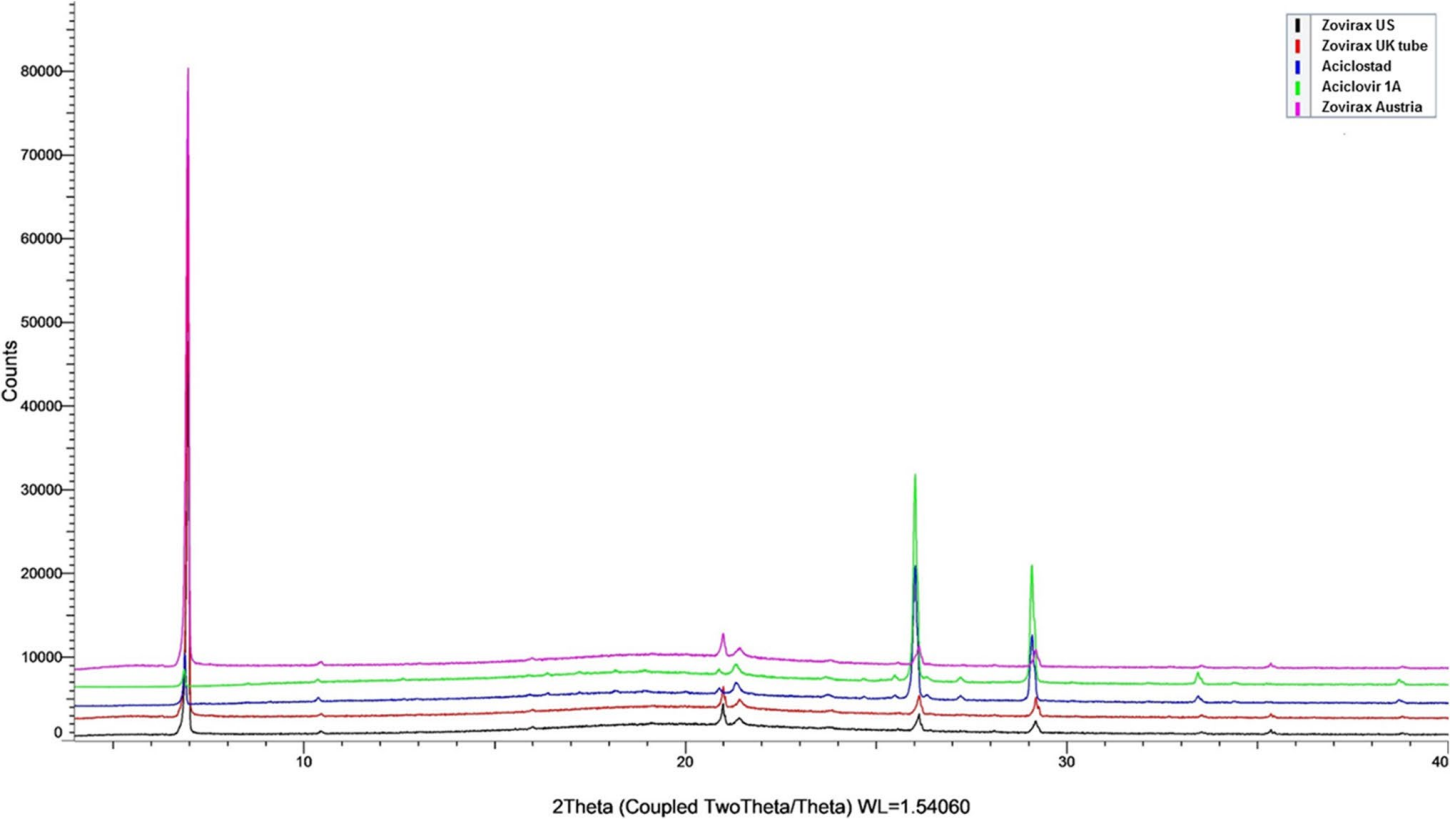
XRPD of acyclovir in a reference and test set of acyclovir cream, 5% products.

X-ray diffractograms of acyclovir in the reference set of Zovirax**®** and in the test set of products are shown in Fig. 4. The drug was crystalline in all cases as it showed characteristic diffraction peaks at 2θ 6.28^°^, 10.46^°^, 16^°^, 21^°^, 22.90^°^, 26.15° and 29.2^°^ [20]. The test set of creams, however, showed low intensity peaks at 6.28^°^, 26.15° and 29.2^°^ suggesting differences in crystal habit/shape between the reference set and test set of products (Table 3).

**Table 3:** Relative intensities of acyclovir peak in acyclovir cream, 5% products in XRPD

2Ѳ	Zovirax® US	Zovirax® UK	Zovirax® Austria	Aciclostad®	Acyclovir 1A pharma®
	**Relative Intensity**				
6.28	33,000	48,000	48,000	4500	1500
26.15	2000	2000	2000	21,000	13,500
29.2	2000	2000	2000	7100	12,500

**Fig. 5:**
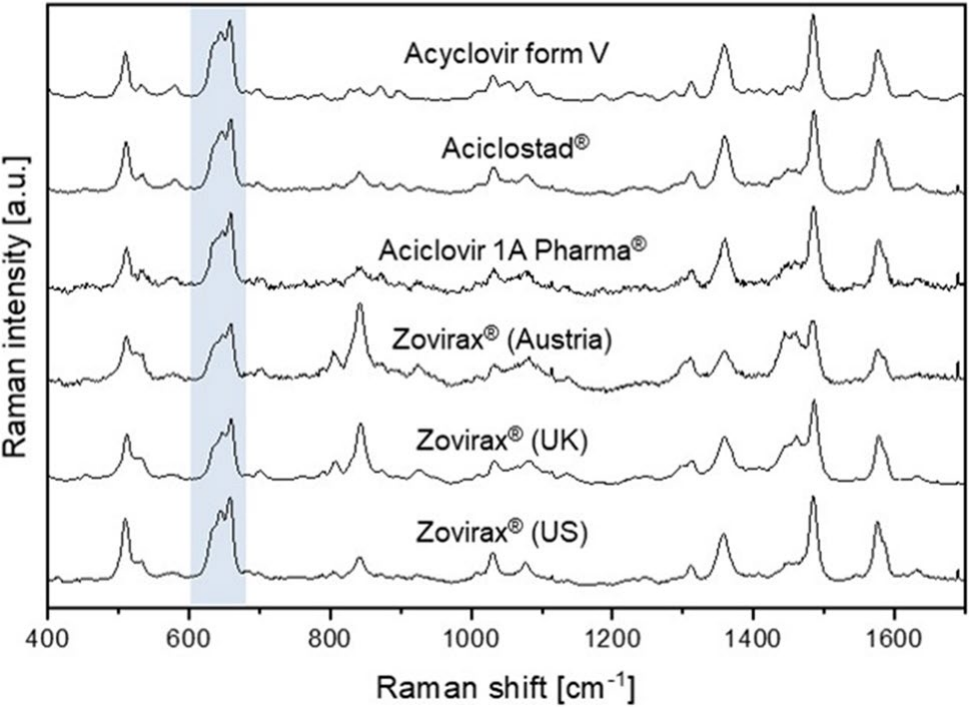
Identification of polymorphic form in the reference set of Zovirax® cream products and the test set of cream products. (**A**) Raman spectra of acyclovir crystals were recorded in all formulations and polymorphic identity was determined by comparison to reference spectra of the five polymorphic forms [20].

Polymorphism of the API was further analysed with CRM. Raman spectra of acyclovir crystals were acquired and compared to reference spectra of all polymorphic forms to determine the polymorphic identity of the API in the reference set of Zovirax® cream products and the test set of cream products (Fig. 5).

The characteristic peaks in the region between 600 – 700 cm^−1^ (highlighted in Fig. 5(A)) were used for the determination of the polymorphic state. The commercially available form V of acyclovir was identified in all formulations.

DSC was used to evaluate the physical properties of the API in the cream base and compare the DSC profiles between the reference set of Zovirax® cream products and test set of cream products. The DSC curve of acyclovir reference showed an endothermic event between 245–255 °C with a melting temperature of 250.2 °C in agreement with the literature values [20]. The DSC curves of the different acyclovir creams (5%) were similar, but a clear endotherm was not evident in between 245—255 °C. This may be due to the physical/chemical interaction of the active with the excipients present in the base.

### Product pH

The pH of the reference set of Zovirax® products was in the range of 6.0—7.5 and found to be higher compared to the test set of products (Table 4).

**Table 4:** pH of Zovirax® and non-Zovirax® products

Name of the Product	pH readings					Average
Aciclostad**®**	4.55	4.52	4.56	4.65	4.57	4.57
Aciclovir A1 Pharma**®**	5.77	5.73	6.06	6.04	5.83	5.89
Zovirax**®** UK	7.39	6.98	6.99	7.25	7.14	7.15
Zovirax**®** Austria	6.93	6.7	6.64	6.93	6.91	6.82
Zovirax**®** US	6.03	6.19	6.71	6.63	6.64	6.44

### Rate of Evaporation of Water from Acyclovir Creams

**Fig. 6:**
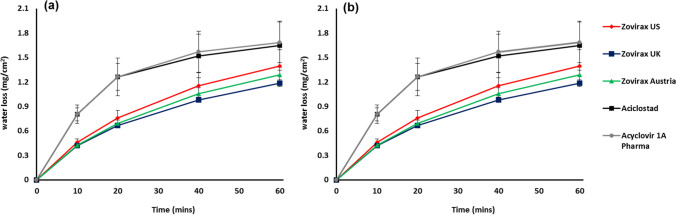
Comparison of water loss by measurement of (**a**) TEWL by AquaFlux and (**b**) weight measurements. Mean of 3 measurements (± 90% CI).

It was observed that the water loss predicted from TEWL for the test set of Aciclostad**®** and Aciclovir 1A Pharma**®** creams was higher than the reference set of Zovirax® products. Substantial differences were not seen in water loss between the Zovirax® creams (Fig. 6). A similar trend was observed for the water loss at rest determined by the gravimetric method.

Detailed comparison of the amount of water lost as predicted by AquaFlux and gravimetric measurement showed that the water loss measurements from these methods are consistent up to 20 min post application. Differences appear after 20 min where the water loss predicted by TEWL was found to be higher compared to gravimetric measurement, but these were not statistically significant.

### Water Content of Acyclovir Creams

The average water content in the creams varied widely. Zovirax**®** products had lower water content than Aciclostad**®** and Acyclovir 1A Pharma**®** (Table 5). These results correlate with the higher rate of evaporation observed in the water loss assessments reported above.

**Table 5:** Water content of Zovirax® and non-Zovirax® products

Product	Average water content
Zovirax**®** UK	25.45
Acyclovir 1A Pharma**®**	60.46
Aciclostad**®**	49.70
Zovirax**®** Austria	27.95
Zovirax**®** US	23.76

### Quantification and Skin Permeation of Acyclovir from Zovirax® and Non-Zovirax® Products

A good linear relationship was observed between the acyclovir peak area and concentration for calibration standards from 0.048 to 300 µg/mL, with a high correlation coefficient (r^2^ = 0.9998). The HPLC method was precise (intra- and inter-day variation was < 5.0%) and accurate (mean recovery 99.5%). The lower limit of quantification for acyclovir was 5 ng/mL.

**Fig. 7:**
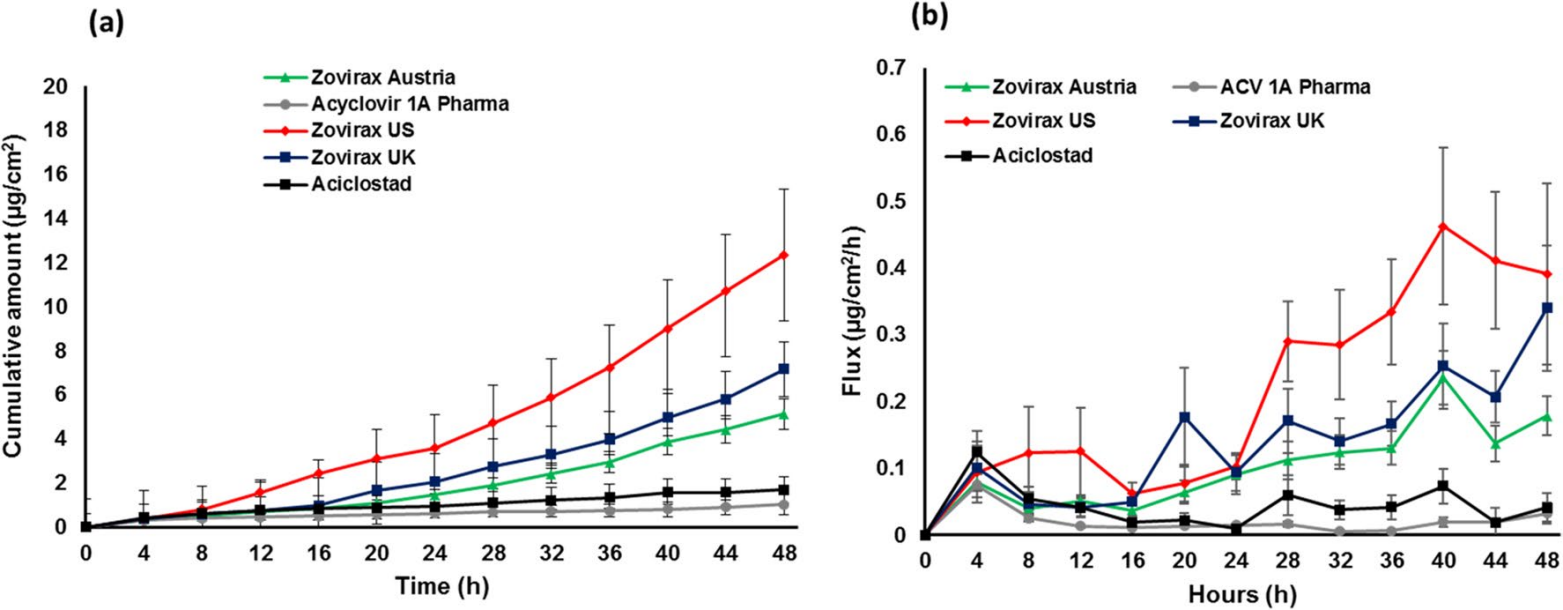
(**a**) Permeation profile and (**b**) flux curve of acyclovir from a reference set of Zovirax® acyclovir cream, 5% products and a test set of acyclovir cream, 5% products across heat separated human epidermis. Results are expressed as mean ± SEM, 3 skin donors, n = 3.

The IVPT study was undertaken using static Franz type diffusion cells to compare the rate and extent of BA of Zovirax® US acyclovir cream, 5% with other Zovirax® acyclovir cream, 5% products available in different parts of the world, and the two other Austrian acyclovir cream, 5% products, Aciclostad**®** and Acyclovir 1A Pharma**®**. Figure 7 (a) illustrates the cumulative amount of acyclovir permeated across heat separated human epidermis over 48 h. Acyclovir permeation from Zovirax® US acyclovir cream, 5% was highest followed by Zovirax® acyclovir cream, 5% products from the UK and Austria (12.35, 7.16, 5.13 µg/cm^2^ for Zovirax® US, UK, and Austria respectively: mean ± SEM). Acyclovir permeation was lowest from the test set of products. The cumulative amount permeated and flux of acyclovir from Aciclostad® and Acyclovir 1A Pharma® acyclovir cream, 5% products was significantly lower (p < 0.05) than the reference set of Zovirax® acyclovir cream, 5% products. Figure 7 (b) illustrates the flux of acyclovir calculated across consecutive sampling points, over the 48 h.

## Discussion

The characterization of CQAs in specific topical semisolid products is an important aspect of product and process understanding for prospective generic products, and for the development of all topical products [23]. Recent progress has also been made on supplementing *in vitro* performance testing and Q3 characterization data with modelling and simulation based approaches [24, 25].

In this work, we have identified several CQAs that are likely to be important when characterising topical semisolid products, although the relevant CQAs will depend upon the product. We have also developed a toolkit of test methods by which to characterize these attributes. We have illustrated that when the profile of CQAs is well matched, then so is the product performance in terms of the rate and extent of topical bioavailability. For the set of acyclovir cream, 5% products evaluated in this study, we were able to identify specific CQAs that appeared to influence the rate and extent of BA for these creams [5].Since compendial test methods have not yet been established for the characterization of relevant CQAs in topical semisolid products the methods developed and described here are of great utility to academia, industry (the pharmaceutical and personal care industries), and regulatory agencies.

Our choice of acyclovir cream, 5% products for developing our toolkit was based on the availability of sets of test and reference products from different manufacturers, and the previous data in scientific literature suggesting the importance of CQAs in these products [9]. Trottet et al. [17] reported that different pharmaceutically equivalent acyclovir cream, 5% products exhibited substantial differences in relative BA when compared in an IVPT study. They attributed the differences between the sets of products to formulation differences, and specifically to differences in the amount of PG present in the different products, but did not elucidate the mechanism by which compositional differences between product formulations can alter specific properties of the physicochemical environment and the structural attributes that collectively govern the performance of such creams.

In this study the cumulative amount of acyclovir permeated across heat separated human epidermis was significantly higher from the reference set of Zovirax® products compared to the test set of products, Acyclovir 1A Pharma® and Aciclostad® (Fig. 7). This observed difference between the test and reference sets of products may arise predominantly from a difference in PG content [26]. From the published literature and publicly available information, Zovirax® products are indicated to contain a relatively higher percentage of PG compared to the other creams (reportedly approximately 40%) [4, 17, 27]. As PG is a fatty alcohol which functions as a co-solvent and a penetration modifier, the higher PG in the Zovirax® products may contribute to the higher acyclovir skin permeation seen in our and previous studies [17, 26]. However, we also showed that whilst Zovirax® products from the UK and Austria displayed similar acyclovir permeation compared to each other (Fig. 7), the cumulative amount and flux of acyclovir across the skin was highest for Zovirax® US. This suggests that whilst PG content may be contributing to the performance differences, other factors (CQAs) also appear to be important in determining product performance. These were systematically assessed, and their influence on acyclovir skin permeation was evaluated.

The first structural feature evaluated was acyclovir particle size and shape, with two-dimensional particle projected area diameter determined by optical microscopy. The differences in acyclovir particle size in the different formulations may be attributed to the sourcing of the active ingredient by different manufacturers. All Zovirax® products evaluated exhibited a thin plate-like crystal morphology, whereas the test set of products from Austria were more irregular in shape. CRM was used as a complementary technique and provided similar sizing and morphological information.

To develop our toolkit, we also utilized optical profilometry and AFM, with both techniques confirming the particle size and shape information (images not included). Optical profilometry (contact and non-contact) also enabled us to visualize deep inclusions in the complex cream microstructure that were not clearly visible on the surface. We found optical profilometry using a LEXT Olympus system to be a useful approach to characterize structural attributes, as the sample preparation was simple and the technique has the potential for large scale automation. The optical profilometry was useful for the preliminary identification of surface microstructural and topographical attributes. In contrast AFM can be challenging for analysis of cream and emulsion-based samples where the size of the inclusions may be too large to map using AFM, and the technique cannot be performed on samples that have not been dried. Whilst it may be useful for analysing smaller inclusions which are not visible with optical microscopes (typically using a 100X magnification), the sample preparation and instrument handling require highly specialized skills, and the cost of carrying out multiple sets of analyses may be prohibitive. Based on our experience, optical microscopy offers the simplest, fastest, and cheapest method for particle sizing and for simultaneously assessing the shape of drug crystals suspended in topical semisolid dosage forms.

The internal microstructure of the products in this study was visualised using cryo-SEM to capture images of any globules present and to further explore the fabric of the matter in the creams. This technique, and the detailed protocol developed for preparing the cream samples, effectively maintained the cream microstructure in its native state without causing gross structural destruction. Features that were not clearly identifiable using optical microscopy or CRM (due to limited resolution) such as nano-meter sized globules in the test set of products, Aciclostad® and Acyclovir 1A Pharma® were clearly evident using cryo-SEM. Moreover, the lamellar arrangement of surfactants in the Zovirax® products was clearly visible when using cryo-SEM.

Where CRM provided advantages was in the spectral identification of the components present in the different creams. The method showed a cream base that was homogeneous (at least, within the limits of the resolution of the microscope) in all the Zovirax® products. CRM also identified PG and paraffin to be the components mainly responsible for the Raman spectra of the cream base of the different products [28].

The pH of a topical semisolid product can influence the solubility of the drug, and thus the amount of drug in dissolved molecular form that is available for diffusion and partitioning into the skin. The solubility of acyclovir is 2–4 mg/ml within the pH 2–9 range. The concentration of acyclovir in all the products is higher than the saturation solubility of acyclovir, therefore most of the acyclovir present is suspended in the cream base either in the aqueous or oleaginous phases. Aciclostad® and Acyclovir 1A Pharma® had a lower pH compared to the Zovirax® set of products (Table 4). This difference is attributed to the composition of the products, and more specifically, to the surfactants employed in the different formulations. The order of addition of these surfactants may also influence the final product pH. Acyclovir is predominantly present in a cationic form in the Austrian test set of products whereas the anionic species dominate in the reference set of Zovirax® products. The pH 5.89 of Acyclovir 1A Pharma® demonstrates the prevalence of zwitter ionic species and Shojie et al. [29] reported that the minimal permeability coefficient of acyclovir was at a pH of 5.8. Although it may be one of the possible causes of lower skin permeation of acyclovir from this product, we cannot draw a direct relationship between the pH difference and the differences in *in vitro* skin permeation of acyclovir from the different creams since the products are not compositionally equivalent and differences in the inactive ingredients can contribute substantially to the *in vitro* transport of acyclovir across human epidermis. Also, the percent of acyclovir in the un-ionized form is similar across the pH range of 4–9.

The evaluation of crystal structure, polymorphism, and solvate form is an important pre-formulation activity. Polymorphs differ in melting point, solubility and processing behavior [30]. Polymorphs in the acyclovir creams were characterized by XRPD, DSC and CRM. CRM identified the polymorphic form V of acyclovir in all formulations [28].XRPD confirmed the acyclovir crystalline nature (Fig. 4) and showed low intensity peaks at 2 theta values of 6.28, 26.15 and 29.2 in the test set of creams. This may be attributed to the dissimilarities in crystal morphology between the test set of products (irregular shaped crystals) and the reference set of Zovirax® products (regular thin plates). The DSC curves were similar for all products with no clear endotherm for acyclovir between 245—255 °C possibly due to the physical/chemical interaction of the active with the excipients present in the base. These results demonstrate the utility of XRD and CRM as the preferred techniques to characterize the polymorphic form of the drug in a topical semisolid product.

An important attribute that may affect product performance following application to the skin is the amount of water present in a topical cream. Water can influence acyclovir solubility in the product and in the skin, and may thereby serve as a penetration modifier for acyclovir [31]. Product drying is related to both the amount of water present in the formulation and the rate of evaporation. Characterization of these attributes is particularly important when conducting skin permeation experiments for longer durations, as this may lead to evaporation and concomitant drying of the cream upon the skin. Karl Fischer analysis of the creams revealed a higher amount of water present in Aciclostad® and Acyclovir 1A Pharma® creams than the Zovirax® set of products. To further understand the rate of evaporation of water from the creams, we designed a novel method by using an AquaFlux meter that is routinely used to determine the TEWL from skin. The method was validated by standard gravimetric measurements. We showed that the evaporative water loss from the test set of products was higher over a 60 min duration compared to the reference set of Zovirax® products (Fig. 6). A similar trend was observed using standard gravimetric measurements, although a larger variability was encountered in the measurements after 20 min, which may be associated with differences between the two techniques.

We hypothesize that a higher rate of water evaporation from the test set of creams may result in a relatively more rapid drying of the test set of products on the skin compared to the reference set of products, leading to more rapid crystallization of acyclovir and a corresponding decrease in the amount of solubilized drug available for diffusion and partitioning into the skin. This mechanism could contribute to the relatively lower amount of acyclovir permeating into the skin from the test set of products compared to the reference set of Zovirax® creams; it also highlights the importance of solvent content and evaporation rate as critical attributes that can substantially influence the BA and BE of topical products. It should be noted that in the reference set of Zovirax® products, a relatively lower evaporative loss of water combined with a reportedly higher amount of propylene glycol may both contribute to the relatively greater rate and extent of acyclovir permeation and flux across the skin seen in the IVPT data for the reference set of products compared to the test set of products.

A notable feature of this work was that CQAs were assessed at a physiological skin surface temperature thus simulating “*in use*” conditions. These CQA characterizations included evaporation rate, particle size, and microstructure, all of which were assessed under conditions simulating the typical stresses and strains the cream is likely to undergo during topical application by patients, *in vivo*.

Some of the methods developed and described here have been used previously as quality control and product development tools. Here, for the first time, their utility as a tool-kit to systematically assess and mitigate the risk of potential failure modes for bioequivalence is illustrated. These potential failure modes for bioequivalence may arise from differences between otherwise closely matched products, and this toolkit of methods with which to characterize the arrangement of matter in semisolid dosage forms establishes a novel approach by which to support a demonstration of bioequivalence – one that is based upon comparative product characterizations that can demonstrate whether or not a generic product is as closely matched to a reference product as batches of the reference product are matched to each other. This seminal work provided some of the first results of what has become a substantial body of corroborating evidence that supported regulatory recommendations for using comparative Q3 characterization to support efficient demonstrations of bioequivalence [8]. A detailed description of how the pieces of the puzzle fit together and a detailed and mechanistic understanding of the implications towards product failure has been presented here. Additionally, methods have been described elaborately, encouraging replication in laboratories across academia, industry and contract research laboratories.

## Conclusions

In conclusion, we have developed a toolkit of techniques and protocols for the evaluation of CQAs that influence the performance of topical drug products. We have demonstrated their utility for evaluating a test and reference set of acyclovir creams from different manufacturers, elucidating relevant CQAs that influence the permeation of acyclovir into the skin. The insights gained about how the components and composition of a product, and the manufacturing process, modulates the Q3 attributes which govern the rate and extent of BA can guide the development of topical formulations, help to optimize manufacturing process parameters and controls, and support an assessment and demonstration of BE for prospective generic topical semisolid products.

## Data Availability

All data generated or analysed during the study are included in this article.

## Abbreviations

API: Active pharmaceutical ingredient
AFM: Atomic force microscopy
BA: Bioavailability
BE: Bioequivalence
CRM: Confocal Raman microscopy
CQAs: Critical quality attributes
DSC: Differential scanning colorimetry
HPLC: High-performance liquid chromatography
IVPT: *In Vitro* Permeation Test
PK: Pharmacokinetics
Q3: Physicochemical and structural
PG: Propylene glycol
QAs: Quality attributes
SEM: Scanning electron microscopy
TEWL: Trans-epidermal water loss
XRPD: X ray powder diffraction
